# Long head BIceps TEnodesis or tenotomy in arthroscopic rotator cuff repair: BITE study protocol

**DOI:** 10.1186/s12891-016-1230-5

**Published:** 2016-08-30

**Authors:** Derek Friedrich Petrus van Deurzen, Vanessa Antoinet Bernice Scholtes, Nienke Willemien Willigenburg, Navin Gurnani, Lukas Pieter Eduard Verweij, Michel Pieter Jozef van den Bekerom, BITE collaboration group, Erik E. J. Raven, Jacco A. C. Zijl, Kiem G. Auw Yang, Ronald N. Wessel, Nienke Wolterbeek, Maaike P. J. van den Borne, Koen L. M. Koenraadt, Ron Onstenk, Loes W. A. H. van Beers, M. Krijnen, Hans E. de Meijier, Yde Engelsma, Arne Heneweer, Lijkele Beimers, John Cheung, Inger Sierevelt, W. J. Willems, Max Hoelen, Nina M. Mathijssen, Arthur van Noort, Tjarco D. W. Alta, Jeanette Verhart, Jacco M. G. T. Jenner

**Affiliations:** Department of Orthopaedic Surgery, Joint Research, OLVG, Oosterpark 9, 1090 HM Amsterdam, The Netherlands

**Keywords:** Rotator cuff, Long head biceps tendon, Arthroscopy, Functional result, Randomised controlled trial

## Abstract

**Background:**

Optimal treatment of the diseased long head of the biceps (LHB) tendon during rotator cuff repair remains a topic of debate: tenotomy or tenodesis. A recent meta analysis revealed no difference in strength or functional outcome between treatments. The included studies varied in methodological quality, and only two were randomized controlled trials (RCTs). As strong evidence in favor of either tenotomy or tenodesis is still lacking, we designed this randomized controlled trial to compare functional outcomes after tenotomy and tenodesis when performed in adjunct to arthroscopic rotator cuff repair.

**Methods:**

Patients older than 50 years with a supraspinatus and/or infraspinatus tendon rupture sized smaller than 3 cm, who are encountered with LHB pathology, will be randomized to either LHB tenotomy or LHB tenodesis. Clinical and patient-reported data will be collected pre-operatively, 6 weeks, 3 months and 1 year after surgery.

Primary outcome is overall shoulder function evaluated with the Constant score at 1 year after surgery. As additional measures of shoulder function, two patient reported outcomes (the Dutch Oxford Shoulder Test and the Disabilities of the Arm Shoulder and Hand questionnaire) will be assessed. Other evaluations include cosmetic appearance evaluated by the “Popeye” deformity, elbow flexion strength, arm cramping pain, MRI-based location of the biceps tendon, quality of life, and duration of surgery. To detect non-inferiority with a one-sided, two-sample t-test with 80 % power and a significance level (alpha) of 0.025, the required sample size is 98 patients.

**Discussion:**

Treatment of LHB tendon lesions is performed differently around the world and meta analyses do not provide conclusive evidence in favor of one of these treatments. This study will strengthen evidence on the risks and benefits of LHB tenotomy and tenodesis in adjunct to a rotator cuff repair, which is important for managing patient expectations.

**Trial registration:**

Dutch Trial Register (NTR3255) January 12, 2012, ClinicalTrials.gov (ID NCT02655848) January 14, 2016, retrospectively registered.

## Background

During arthroscopic rotator cuff repair, pathologic lesions of the long head of the biceps tendon are frequently encountered. Controversy revolves around optimal treatment for these lesions. Partial tears involving less than 25 % of the biceps tendon can be treated conservatively with partial debridement or observation. However, when the biceps tendon shows subluxation or a tear greater than 30 %, treatment such as tenotomy [1–3] or tenodesis [4, 5] is considered necessary. With tenotomy, the origin of the long head of the biceps (LHB) is released at its junction with the superior labrum, using a cautery device or curved scissors [1, 3].

LHB tenodesis can be performed in several ways. Generally, it is started by releasing the biceps tendon at its origin and whip stitching the proximal end of the released biceps tendon. Next, fixation in the bicipital groove is obtained using either an arthroscopic interference tenodesis or suture anchoring technique [6].

There are several proposed advantages and disadvantages of the LHB tenotomy as compared to the LHB tenodesis. Proposed advantages of tenotomy are: 1) more time efficient, 2) more cost effective, 3) shorter rehabilitation time, and 4) safer in terms of complications. Proposed disadvantages of tenotomy are: 1) a higher risk for cosmetic deformity called the Popeye sign of the upper arm, 2) risk for loss of elbow flexion and supination strength, and 3) fatigue discomfort. Lack of high levels of evidence on these proposed (dis)advantages limits our ability to recommend LHB tenotomy over tenodesis, or vice versa.

In a recent meta-analysis on treatment of biceps tendon pathology, Gurnani et al. [7] reported no difference in functional outcome and elbow strength between treatments, based on nine original research studies [4, 8–15]. For elbow strength, the included studies consistently reported no difference between treatment options. However, the findings on Constant scores (functional outcome) were more variable, and tended to favour tenodesis (*p* = 0.07). Only the study with lowest methodological quality reported a mean difference in Constant score that tended to favor tenotomy. The meta analysis further confirmed that a Popeye phenomenon occurred more frequently with biceps tenotomy. Arm cramping pain was also more common after tenotomy. It is important to note that the included studies strongly differed in methodological quality, with Coleman scores ranging from 45 to 100, and only two studies were RCTs. In the study by de Carli et al. [12] 65 patients were included. As this is a relatively small number of patients and no power analysis was performed, this bears the risk that no difference can be demonstrated between the treatments. Also subjective assessment of the presence of a Popeye phenomenon by the patient was not performed.

In the RCT conducted by Zhang et al. [15] also large size rotator cuff tears were included. As large size tears have a worse prognosis this may lead to worse Constant scores. The LHB tenotomy was performed as close to the glenoid as possible, possibly creating a whale tail deformity which may lead to an autotenodesis effect, as this may prevent the tendon to retract out of the glenohumeral joint, this may explain different results with regard to occurrence of the Popeye phenomenon. Also, two other recent meta-analyses reported (at least partly) different results [16, 17].

Therefore we designed this RCT and only include small to medium size supraspinatus tears and added patient-reported assessment based on a sample size calculated with a power analysis in an effort to increase the quality of the evidence.

Importantly, self-reported outcome measures are underrepresented in meta-analyses comparing LHB tenodesis and tenotomy. While clinical outcomes assessed by a clinician provide valuable information on recovery after surgery, the patient’s perspective may be even more important. Often, clinician-reported and patient-reported outcomes will correlate, but this is not always the case. For instance, a Popeye deformity reported by a clinician may not be noticed by patients, and often does not bother them [8]. To determine the clinical relevance of previously reported findings, it is critical to consider both patient’s and the clinician’s perspectives. In other words, clinical measures (e.g. range of motion, strength, MRI) should be complemented with patient-reported outcomes (e.g. self-reported function, pain, cosmetic appearance, and quality of life).

Therefore, we designed this multicenter, prospective randomized controlled trial that aims to compare the functional results between LHB tenotomy and LHB tenodesis when performed during arthroscopic rotator cuff repair. The primary outcome measure of this trial is the Constant Score at 1 year follow-up. Secondary objectives are to compare elbow flexion strength, Popeye deformity, and patient reported function and quality of life between intervention groups. In addition, we aim to quantify the relation between clinician-reported and patient-reported function and cosmetic appearance (Popeye phenomenon) in our population. Based on the largest meta-analysis so far [7], we hypothesize no difference in functional outcome after tenotomy compared to tenodesis, as reflected in similar Constant scores at 1 year follow up.

## Methods

### Trial design

A prospective patient blinded randomized controlled multicentre trial, with parallel groups will be performed at 11 hospitals in the Netherlands: Gelre Ziekenhuizen, Apeldoorn; St. Antonius Ziekenhuis, Utrecht; Amphia Ziekenhuis, Breda; Groene Hart Ziekenhuis, Gouda; Onze Lieve Vrouwe Gasthuis, Amsterdam; Medinova, Rotterdam; MC Slotervaart, Amsterdam; Delairessekliniek, Amsterdam; Reinier de Graaf Gasthuis, Delft; Spaarne Ziekenhuis, Hoofddorp; Canisius Wilhelmina Ziekenhuis Nijmegen. Inclusion will be competitive. The allocation ratio between the two interventions will be 1:1 and a non-inferiority design will be used.

This trial is registered at the Dutch Trial Registry on Jan 18, 2012, file number NTR3255, and at ClinicalTrials.gov on Jan 14, 2016, ID number NCT02655848.

### Participants

The study population will consist of patients older than 50 years who are indicated to undergo repair of a supraspinatus and/or infraspinatus tendon rupture sized smaller than 3 cm and who are encountered with an inflamed, unstable or partially torn LHB tendon. All patients who meet the inclusion criteria are approached for participation in the BITE study.

#### Inclusion criteria

- Patients older than 50 years
- Full thickness degenerative rotator cuff tear of supraspinatus/infraspinatus tendon, smaller than 3 cm (measured at the time of surgery using an arthroscopic ruler).
- Patients need to be able to read and write in Dutch language in order to complete the questionnaires, and sign informed consent.

#### Exclusion criteria

- Traumatic-, or partial thickness rotator cuff rupture
- Full thickness tear larger than 3 cm measured using an arthroscopic ruler.
- Accompanying subscapularis tendon lesion
- Hourglass deformation biceps tendon origin or accompanying subscapularis tendon rupture.
- Osteoarthritis of the glenohumeral joint, defined as narrowing of the glenohumeral joint space or osteophytes, using AP X-ray of the affected shoulder
- Acromion to humeral head distance measuring 6 mm or smaller, defined by Hamada classification as grade 2 or higher.
- Prior surgery to the involved shoulder
- Dementia or inability to complete questionnaires and assessments

### Study procedures (patient flow)

#### Enrolment

The treating physician/investigator will approach potential participants about the study during initial visit in the office. The study will be described in detail and the informed consent form will be given for patients to read. It will be emphasized that participation is voluntary. After obtaining a signed informed consent document, all patients selected for arthroscopic repair of a ruptured infraspinatus or supraspinatus tendon or both will be assessed preoperatively. Definite inclusion will be determined during surgery: in case significant biceps pathology is found during arthroscopic surgery the patient will be randomized to Group 1 or Group 2 (see below: Randomization and blinding).

#### Pre-operative assessment

At the initial visit, the treating physical will determine the Constant score, cosmetic appearance, elbow flexion strength and results of MRI. In addition, all subjects will complete a questionnaire with demographic information, questions on general pain and pain in the bicipital groove, and different patient reported outcome measures, such as pain on numerical reporting scale, DASH, DOSS, EQ-5D and a questionnaire assessing cosmetic appearance. An MRI and digital photograph are taken from the upper arm to define a baseline for assessing cosmetic changes after surgery. Using the MRI we will identify whether the LHBT is located in de bicipital groove and evaluate integrity of the rotator cuff repair (Table 1).

**Table 1:** Study overview

Main outcome measure	Pre operative	6 weeks	3 months	1 year
Constant Score	X		X	X
Patient reported				
DASH	X	X	X	X
DOSS	X	X	X	X
Cosmetic appearance	X	X	X	X
Pain	X	X	X	X
EQ-5D	X	X	X	X
Clinician reported				
Cosmetic appearance	X	X	X	X
Elbow flexion strength	X		X	X
MRI	X			X

Legend: *DOSS* Dutch Oxford shoulder score, *DASH* Disabilities of arm, shoulder and hand

#### Post-operative assessments

The study includes three post-operative assessments; at 6 weeks, 3 months, and 1 year after surgery. Table 1 shows which data are collected at each of the follow up visits. The Constant score and strength testing is not performed at 6 weeks postoperatively, in order not to jeopardize the rotator cuff repair.

### Randomisation and blinding

Randomisation occurs in the operation room. Only when significant biceps pathology is found (during arthroscopic surgery), and the size of the rotator cuff rupture does not exceed 3 cm, the patient will be randomly allocated to a tenodesis or tenotomy. If one or both of these criteria are not met, the surgeon continues with his/her treatment of choice.

Randomisation is performed at a secured website that the local surgeon can only access via a personal login code and password. Randomization occurs in a 1:1 ratio by a computerized program (TENALEA Clinical Trial Data Management System), using random blocks with maximum block size 6, stratified by centre.

After definite inclusion and enrolment in this trial (during surgery, see below), patients will be assigned an anonymous study identification number. Only the study identification number will be used on data forms and in the databases. The encryption between study identification number and personal information will only be accessible for the research coordinator, the research assistant, and PI of this trial.

All patients will be blinded for the type of treatment (LHB tenodesis or tenotomy) for the duration of 1 year. Data will be processed and analysed by a blinded investigator. After finalising data analyses the blinding will be broken for publication purposes.

### Interventions

#### Arthroscopic LHB tenotomy

Patients randomized into the LHB group will undergo a tenotomy in which The LHB tendon is released form its origin at the superior labrum.

#### Arthroscopic LHB tenodesis

Patients randomized into the LHB group will undergo a tenodesis in which the LHB tendon is released form its origin and loaded with a suture. Subsequently, the LHB tendon is tenodesed in the bicipital groove using a bio interference technique or with the remaining sutures of the anterior most suture anchor that is used for the rotator cuff repair. All participating surgeons are experienced shoulder surgeons and have performed these procedures at least 20 times.

#### Rehabilitation

Because the patients are blinded to their treatment, they all follow the same postoperative rehabilitation instructions. In the first 6 weeks postoperatively, patients wear a sling and only passive range of motion exercises are allowed under guidance of a dedicated shoulder physiotherapist. After 6 weeks, active movement is started and expanded gradually. Patients are not allowed to lift any objects heavier than one kilogram during 3 months.

### Outcome measures

#### Primary outcome

The primary outcome measure is the Constant score at 1 year follow up.

The Constant score is a 100 points scale consisting of four variables, which includes a patient reported part (pain 15 points and activity level 20 points), which results in a total of 35 points. Next to this there is a physician rated part (shoulder strength 25 points and range of motion 40 points) which totals 65 points [18]. As scores of the Constant-Murley test are gender related and will decrease with age, we will normalize the summed scores according to Katolik et al. [19].

#### Secondary outcomes

Secondary outcomes consist of Patient Reported Outcome Measures (PROMS), Clinician Reported Outcome Measures (CROMS), clinical assessments, and imaging assessments.

#### PROMs

Patient-reported shoulder function will be measured with the Disabilities of Arm, Shoulder and Hand questionnaire (DASH) [20], and the Dutch Oxford Shoulder Score (OSS) [21]. The DASH is a self-report questionnaire (scale 0–100) designed to measure physical function and symptoms in patients with musculoskeletal disorders of the upper limb. A higher score indicates more disability. The OSS (scale 0–48) is a self-reported questionnaire focussing on shoulder complaints experienced in the past 6 months, 3 months and 4 weeks. A lower total score indicates more disability.

**Fig. 1:**
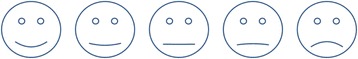
Smiley scale. Legend: Indicating patient satisfaction with cosmetic appearance of their operated arm

Patient reported cosmetic appearance will be measured by the presence of a Popeye sign (yes/no), as well as the cosmetic appearance on a smiley scale (Fig. 1).

Patient reported pain will be recorded for pain in general and for pain in the bicipital groove. For both types of pain, the incidence will be evaluated (yes/no), as well as the level of pain on a numeric rating scale (NRS, 0–10). Patient reported quality of life will be assessed using the EQ-5D [22, 23]. This questionnaire covers five domains (mobility, personal care, daily activities, pain, and mood), as well as a 100-point thermometer on general health. Each domain has three response categories; no problems, moderate problems or severe problems. Crosswalk value sets for the Dutch population will be used to quantify health state with a value between −0,56 and 1, with 1 representing the best imaginable health state.

#### CROMs

Clinician reported cosmetic appearance will be measured by the presence of a Popeye sign (yes/no). In addition, the clinician will make a digital photograph of the biceps, using a standardised protocol. These digital pictures will also be scored by blinded, independent assessors, both on presence of a Popeye sign (yes/no), and cosmetic appearance on a smiley scale (0–5).

#### Clinical assessment

To evaluate elbow flexion strength, the Elbow Strength Index (ESI) will be calculated. The ESI is calculated by dividing elbow flexor power with the lower arm in full supination by that of the contralateral upper extremity using a dynamometer (www.idorth.com) Innovative Design Distributors, London, UK [10, 24].

#### Imaging

Postoperative MRI, after 1 year is used to assess location of the proximal biceps tendon with regard to the bicipital groove. Absence of the biceps tendon in the bicipital groove confirms a successfully performed LHB tenotomy. Absence of the biceps tendon in the bicipital groove confirms failed LHB tenodesis. The rotator cuff is classified as fully healed, partially healed or recurrent rupture. Also the quality of the rotator cuff is scored according to Patte and Goutallier [25, 26].

#### Duration of surgery and complications

Surgeons will report the start and end times of their tenotomy and tenodesis procedures, so the duration of surgery can be calculated. In addition, all complications reported by patients up to 1 year follow up will be registered.

### Sample size

Sample size calculations are based on the Constant score, to evaluate the primary hypothesis: LHB tenotomy does not lead to inferior functional results at 1 year postoperative than LHB tenodesis when performed in conjunction with an arthroscopic repair of a moderately sized supraspinatus/infraspinatus tear in patients 50 years or older.

In 2013 Kukkonen et al. [27] presented results on minimal important change in rotator cuff surgery. Based on these data we performed a calculation of the sample size. Anticipating a difference of less than ten points on the Constant Score and a standard deviation of 16 points in both groups, we calculated the sample size using Study size 2.0 Software. To detect non-inferiority using a one-sided, two-sample t-test with an 80 % power and a significance level (alpha) of 0.025, group sample sizes of 41 patients are needed. Anticipating a dropout rate of 20 %, a total of 98 patients will be included. METC approval Nov 18, 2014 (NL 37898.100.11).

### Statistical analysis

Statistical analyses will be done using the Statistical Package for the Social Sciences (SPSS Chicago, Illinois, USA). Analyses will be performed according to the intention to treat principle. To test our main hypothesis that LHB tenodesis and tenotomy result in similar functional outcome at 1 year follow up, Constant scores will be compared between groups using an independent samples t-test. In the unlikely event that a baseline difference is observed between groups, the change in Constant score from baseline to 1 year post-surgery will be used for analysis. Given the non-inferiority design, a one-sided *P*-value <0.025 will be considered statistically significant. Statistical uncertainties will be quantified using 95 % two-sided confidence intervals.

To further compare recovery after the two surgical interventions, longitudinal data analysis (mixed model or generalized estimating equations (GEE)) [28] will be used. In the primary model, the Constant score will be included as the dependent variable, and treatment allocation (between subjects) and time (within subjects) will be included as the key independent variables. Other variables that may affect outcome (e.g. age, center of inclusion) will be included as covariates. The interaction between group and time will be assessed to evaluate whether the two treatment groups differed in change over time. The secondary outcome variables (e.g. shoulder function on the DASH and OSS, quality of life on the EQ5D, pain on the VAS, Elbow strength, MRI findings, and cosmetic appearance) will be analyzed in using similar models. For all longitudinal data analysis analyses, a two-tailed value of *p* < 0.05 is considered statistically significant.

To evaluate the relation between patient-reported and clinical measures of function, we will calculate correlation coefficients for continuous outcome measures (e.g. DASH and OSS questionnaires versus Constant scores and elbow flexion strength). To assess agreement between the presence of a Popeye deformity (y/n) as reported by the patient, clinician, independent observers, and based on MRI, we will calculate Kappa coefficients.

### Data storage

Data will be entered into the Statistical Package for the Social Sciences (SPSS, Chicago, Illinois, USA). After the data entry, paper data collection forms will be stored in an archive. Both paper forms and digital databases will only be accessible by the research coordinator (NW,VS), PI (DVD) and research assistant (LV).

### Steering- and data monitoring committee

No official steering committee or data monitoring committee has been appointed for this study. The following representatives from the participating organizations are involved in the project oversight and control: DVD (Principal Investigator), NW and LV. All study related problems or (serious) adverse events will be discussed with the principal investigator DVD, and researchers VS, NW, LV and MB. SAE’s will be officially reported to the ethical committee. The ethical committee judges whether the safety of the patients is jeopardized and whether the trial can be continued or not. Data entry will be performed by one of the researchers (LV). All entered data will be checked and cleaned (LV and NW) according to the quality handbook of the emgo + institute for health and care research (www.emgo.nl/kc).

## Discussion

With this multicentre, prospective patient blinded randomized level I study we will contribute to elucidating the controversy in the treatment of LHB tendon pathology in arthroscopic rotator cuff surgery. If our main outcome measure, the Constant score, is not lower in the group that underwent tenotomy, this increases the level of evidence to support previously reported findings that functional outcome is similar between treatments. Our thorough assessment of Popeye deformity (by the patient, the treating surgeon, and blinded assessors using digital pictures, complemented with MRI imaging) will reveal how this patient population values cosmetic appearance. While a Popeye deformity is more common after tenotomy, it is to date unknown to what extent this bothers patients older than 50 years.

A limitation of this study is that our comparison of costs is limited to the duration of surgery, without taking into account any other financial aspects. With regard to surgical material, no differences are expected, as tenodesis can be performed with the same anchor that is already used for the rotator cuff repair. One could speculate that tenotomy may allow for earlier return to work or activities, but this potential advantage would be masked by our study design as both groups received the same post-operative rehabilitation instructions.

The results of this study will strengthen the evidence on potential risks and benefits associated with tenotomy and tenodesis. Insight in the differences between treatment effects is very important, as managing patient expectations is critical in current orthopaedic practice.

## Abbreviations

ADL: Activities of daily living
BITE: BIceps tenotomy or tenodesis
DASH: Disabilities of the arm, shoulder and hand questionnaire
DOST: Dutch translation of Oxford shoulder test
EQ-5D: Euroqol-5 dimensions
GCP: Good clinical practice
GEE: General estimated equations
LHB: Long head biceps
METC: Medische ethische toetsings commissie
NVA: Nederlandse vereniging voor arthroscopie
OLVG: Onze lieve vrouwe gasthuis
RCT: Randomised controlled trial
VAS: Visual analogue scale
